# Melatonin induces a stimulatory action on the scrotal skin components of Soay ram in the non-breeding season

**DOI:** 10.1038/s41598-020-67103-5

**Published:** 2020-06-23

**Authors:** Fatma El-Zahraa A. Mustafa, Fatma M. Abdel-maksoud, A. H. S. Hassan, Doaa M. Mokhtar

**Affiliations:** Department of Anatomy and Histology, Faculty of Vet. Medicine, Assiut University, Assiut, Egypt

**Keywords:** Cell biology, Developmental biology, Structural biology, Anatomy

## Abstract

Fifteen adult Soay rams were employed in this study to investigate the effect of melatonin on the scrotal skin using histological, histochemical, and morphometrical analysis. The results revealed that the melatonin treated group showed a significant increase in the thickness of the epidermis, the cross-sectional area of blood capillaries and nerve fibers compared with the control one. In addition, obvious hypertrophy and hyperplasia were detected in the sebaceous glands in association with a significant increase in the number and diameter of apocrine sweat glands with well-developed secretory activity. S100 protein and cytokeratin-19 strongly stained the basal cells of sebaceous glands in the melatonin treated group incomparable to the control group. Moreover, the nerve fibers were intensively immunoreacted for S100 and cytokeratin proteins in the melatonin treated group in contrast to the control one. A high number of telocytes (TCs) could be identified in the treated group around the nerve fibers and blood vessels in the dermis. The number of Langerhans cells showed a significant increase in the melatonin groups that were identified by MHC II and PGP 9.5 within the epidermal layer. Furthermore, a significant increase in the number of dendritic cells was identified in the melatonin group, which were distributed within the dermis, around hair follicles, sebaceous glands, and sweat glands and were strongly expressed PGP-9.5, MHC-II, VAMP, SNAP, keratin-5, and cytokeratin-19 immunoreactivity. Notably, Merkel cells showed a significant increase in the number in the melatonin group that could be stained against nestin, SNAP, and VAMP. On the other hand, the secretory granules in sweat glands were exhibited a strong positive reactivity for synaptophysin in melatonin group. The current study showed that the administration of melatonin induced a stimulatory effect on keratinocytes, non-keratinocytes, sebaceous and sweat glands, hair follicles, as well as the vascular, neuronal, and cellular constituents of the dermis.

## Introduction

Melatonin is a Multitasking hormone that performs several functions to protect the body from different environmental conditions^1^. Melatonin is not only produced by the Pineal gland but also synthesized by the ovary, testes, retina, Harderian gland and skin^2–4^. Melatonin acts as an anti-inflammatory, antioxidant, anti-cancer agent, and exhibited strong anti-aging characters^5,6^.

The skin is considered the largest organ and covered 7–12% of the body and performs several vital functions^7^. The skin is made of two distinguished parts, epidermis and dermis. The superficial one was the epidermis and formed mainly of keratinocytes and non-keratinocytes. Keratinocytes proliferation is regulated by melatonin^8^. On the other hand, the non-keratinocytes including Langerhans cells that act as immune cells (dendritic cells of the epidermis), Merkel cells represent neuroendocrine cells of the epidermis and melanocytes, which are the main source of melanin that responsible for skin pigmentation and protection from ultraviolet rays^9,10^.

The deep part of the skin is the dermis, which consists of connective tissue, fibers and cells such as telocytes, dendritic cells, and fibroblasts. Telocytes are recently described interstitial cells and characterized by small cell body and long cytoplasmic processes^11^. They establish a broad communication with different structural components of the connective tissue^12^. Dendritic cells are antigen-presenting cells with a key role in the initiation of the immune response^13^. The skin appendages include the hair follicles, as well as sweat and sebaceous glands. Interestingly, the hair follicles are important sites of melatonin synthesis and bioregulation^14^. In addition, melatonin receptors MT1 and MT2 were detected on the sweat glands^15^.

Melatonin regulates skin homeostasis and prevents ultraviolet (UV) harmful effect on skin through a melatoninergic antioxidative system^15–19^. Keratinocytes are considered as a target for damage with UV while melatonin protects these cells through the prevention of UV-mediated apoptosis^20–22^. Moreover, the harmful effect of X-ray on the skin is reduced by melatonin^23^.

Our previous works were involved the studying of possible useful effects of melatonin on different organs of Soay rams, which includes Harderian gland^24^, seminal vesicles^25^ and adrenal glands^26^ through the non-breeding season.

There is a lack of a set of data up tell now regarding the effect of melatonin on the skin that represents an essential organ for immunity, aging, and management of different physiological functions. Therefore, the aim of the current study is to investigate the effect of melatonin administration on Soay ram skin including the epidermis, dermis, and their appendages using histological, immunohistochemical and morphometrical analysis.

## Materials and methods

### Experimental design

The experiments were conducted in accordance with the U.K. Animals (Scientific Procedures) Act of 1986 in MRC Reproductive Biology Unit, Centre for Reproductive

Biology, Edinburgh, Scotland, UK. This study was performed in strict accordance with the relevant guidelines and ethical regulations (experiment no. S/17353). The protocol was approved by the Cenral Office for Research Ethics Committees (COREC) in Eastarbourne Terrace, United Kingdom.

According to^26^ briefly, the rams of Soay breed (*Ovis aries*) were obtained from specialist breeders in Scotland. The skin of the scrotum of 15 adult Soay rams (aged 1.5 years) was used in this study. At the end of May, eight animals were given a subcutaneous implant containing melatonin (treated group), while seven animals were given empty implants (control group). The melatonin implants were made of silastic sheeting (500–1Dow Corning, Midland, MI) containing 1 g melatonin (Sigma Chemical, Poole, Dorset, UK), previously shown to produce a constant concentration of melatonin in the peripheral blood in the daylight of about 200–500 pg/mL plasma^27^. The implants were placed subcutaneously above the rib cage using local anesthesia and left in place throughout the experiment. Eleven weeks after the onset of the experiment, all rams were sacrificed by intravenous barbiturate administration, the skin specimens from the scrotum were carefully excised, and small samples were processed for light microscopic examination.

### Histological analysis

The samples from the scrotal skin of both the control and treated groups were dissected and immediately fixed in Bouin’s fluid for 22 h. The fixed samples were dehydrated in an ascending series of ethanol, cleared in methyl benzoate, and then embedded in paraffin wax. Serial sections of 5–8 μm in thickness were cut transversely and longitudinally. The staining protocols and used procedures were carried out following the descriptions of the histological techniques as reported by Bancroft J.D.^28^. The sections were stained using hematoxylin and eosin for demonstrating the general histological structure of the skin and its appendages; Periodic acid Schiff’s (PAS) for demonstrating neutral mucopolysaccharides and Crossmon’s trichrome for the demonstration of muscle fibers and collagenous fibers.

### Immunohistochemistry (IHC)

Paraffin-embedded skin tissue sections (5 μm) were dewaxed, rehydrated, and rinsed 3 times in PBS (pH 7.4) for 5 min. Endogenous peroxidase was blocked by soaking the sections in 1% H_2_O_2_ for 15 min at room temperature^29^, followed by washing. For antigen retrieval, the slides were incubated with L.A.B solution (Liberate Antibody Solution, Polysciences, Inc., USA) for 20 min. Consequently, the sections were incubated overnight at 4 °C with different antibodies (Table 1). The sections were rinsed in 0.2% Triton-X 100/PBS and followed by incubation with a biotinylated secondary antibody (Table 1) for 2 h at RT, Thereafter, the slides were incubated with Vectastain ABC (Avidin-Biotin complex) reagent for 45 min in a humid chamber at room temperature. The detection of the reaction was done with DAB for 5–10 min. The stained sections were observed by Leitz Dialux 20 Microscope and photos were captured by cannon digital camera (Cannon Powershot A95).

**Table 1:** Identity, sources, and working dilution of antibodies used in immunohistochemical and immunofluorescence studies.

Primary antibodies	Primary antibody Supplier (Catalog No.)	Origin	Dilution	Biotinylated secondary antibody (IHC)
1.Anti-S100 protein	Dako, #Z0311	Rabbit polyclonal	1:500 for IHC 1:200 for IF	Goat anti rabbit IgG)
2.PGP 9.5	Sigma immune chemicals# MCA-BH7	Mouse monoclonal	1:2000 for IHC 1:200 for IF	Goat anti mouse IgG)
3.AntiCytokeratin	Sigma immune chemicals# c7159	Mouse monoclonal	1:200	Goat anti mouse IgG
4.SNAP 25	Santa cruz biotechnology (sc-20038)	Mouse monoclonal	1:200	Goat anti mouse IgG
5.VAMP-1/2	Santa cruz biotechnology (sc-20039)	Mouse monoclonal	1:200	Goat anti mouse IgG
6. Anti-Keratin 5	Sigma immune chemicals# SAB4501651	Rabbit polyclonal	1:500	Goat anti rabbit IgG)
7. MHC II	Abcam (ab 23990)	Mouse monoclonal	1:100	Goat anti mouse IgG
8. Anti-Synaptophysin	Sigma immune chemicals# S5768	Mouse monoclonal	1:200	—

IHC staining was quantified within control and melatonin treated groups for Cytokeratin, S100, PGP, MHC II, Nestin, Keratin, VAMP and SNAP using image J software. According to previous studies^30,31^, the color deconvolution algorithm (Aperio) was used to isolate different stains for quantification: the red, green, and blue (RGB) OD color vectors were calculated for each one using default software settings and control slides stained separately with hematoxylin or DAB. To convert the intensity numbers to Optical Density (OD) numbers with the following formula: OD = log (max intensity/Mean intensity), where max intensity = 255 for 8-bit images. This used to quantify the average darkness of the image due to the DAB signal.

### Immunofluorescence (IF)

The sections were dewaxed and rehydrated in a descending series of ethanol then washed with phosphate buffer solution (PBS). Antigen retrieval was L.A.B solution for 20 min at RT. Afterward, the sections were incubated with a blocking solution (PBS containing 5% normal serum), 1% bovine serum albumin (Roth) and 0.3% Triton X-100 at room temperature for 2 h^29^. Then, the sections were incubated either with anti-S100 proteins and PGP 9.5 or with anti-synaptophysin (Table 1). In the following day, the sections were washed in PBS (3×10 min) and incubated 2 h (in dark) with either with Alex Fluor- 488 conjugated anti-rabbit IgG (1:500, molecular probe, OR) and Cy3-conjugated anti-mouse IgG (1:100, molecular probe, OR) or Cy3-conjugated anti-mouse IgG only. Finally, the sections were washed (3 × 5 min each) in 1 X PBS then coverslipped with Vectashield (Vector-USA). Slides were viewed with a fluorescence microscope (Axioskop 2 Plus).

The quantified synaptophysin puncta in the scrotal skin in the control and melatonin groups were performed using image j software. The processing of images and quantification “Quantification was performed on a minimum of three fields from a minimum of 3 separate animals.”

### Morphometrical studies

The morphometric studies were carried out on the immunohistochemical images of the skin of both control and melatonin-treated groups using Image-J software. The measurements were done on 15 randomly selected sections of the skin per animal (5 different areas were measured from each section) as follows:^32^ the number of Langerhans, Merkel cells, keratinocytes, melanocytes and dendritic cells per 50 μm^2^ using × 40 objective. In addition, the.cross-sectional area of blood capillaries and nerve fibers (μm) using × 100 objective, the quantity of the sweet gland per 200 μm^2^ using ×40 objective, the diameter. of the sweat glands (μm) using × 40 objective, and the thickness. of epidermis layer using × 10 objective were measured.

### Statistical analysis

The data of immunohistochemical and immunofluorescence analysis in addition to the morphometrical measurements were summarized in Fig. 14 and Table 2, respectively using “GraphPad Software” (Version 6.05, International Scientific Community) to compare between different variables in both control and treated animals. Differences were considered significant if P < 0.05 (*) and P < 0.01 (**) and highly significant if P < 0.001 (***). All data were statistically analyzed using unpaired t-test.

**Figure 1:**
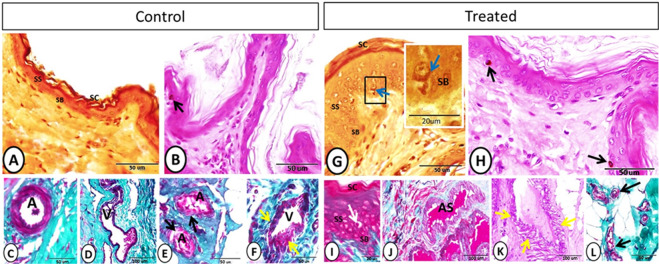
Stratum basale (SB), stratum spinosum (SS), stratum corneum (SC) and vascular elements after the melatonin treatment. (**A–C**) Grimelius silver nitrate method showing the different skin strata and Merkel cell (blue arrow). (**B–H**) PAS-Hx staining showing melanocytes containing dark brown pigment (black arrow). (**I**) Crossmon’s trichrome staining showing Langerhans cell (white arrow) between the cells of stratum spinosum. (**C–D**) Crossmon’s trichrome staining showing artery (A) and vein (V) at the deep position of the dermis. (**E**) Arteries (A) with glomus cell on tunica media (black arrow) (Crossmon’s trichrome). (**F**) A vein showing irregular wide lumen (V) and media of different thickness (yellow arrow). (**J**) Artery of special structure showing wide irregular lumen (AS) (Crossmon’s trichrome). (**K**) Arteries of special structure showing smooth muscle media of different thickness along the wall (yellow arrows) (PAS-Hx staining). (**L**) Glomus cells on the wall of arterioles (black arrows) (Crossmon’s trichrome).

**Table 2:** Morphometric Measurements in Control and Melatonin-Treated Groups.

Measurements	control	After melatonin treatment
The number of Langerhans cells/50 μm^2^	1.12 ± 0.34	4.29 ± 0.49***
The number of sweat glands/200 μm^2^	6.42 ± 1.26	15.25 ± 2.94*
Diameter of sweat glands (μm)	71.99 ± 6.80	98.96 ± 7.30*
The number of Merkel cells/50 μm^2^	2.10 ± 0.50	4.50 ± 0.70**
The number of DCs/50 μm^2^	2.20 ± 0.58	3.80 ± 0.37*
Thickness of the epidermis (μm)	16.66 ± 3.50	29.09 ± 4.84*
The number of keratinocytes/50 μm^2^	15.10 + 2.80	28.0 + 3.20**
The number of melanocytes /50 μm^2^	4.50 + 0.30	5.10 + 0.50 ^NS^
Cross sectional area of nerve fibers (μm)	553.10 ± 99.80	2923.11 ± 675.0**
Cross sectional area of blood capillaries (μm)	343.40 ± 85.85	1430.0 ± 122.60***

The measurements are expressed as the mean ± SE.

***p < 0.001,**p < 0.01, *p < 0.05.

### Ethical approval and consent to participate

Experiments no. (S/17353) were conducted in accordance with the U.K. Animals (Scientific Procedures) Act of 1986.

## Results

### Histological analysis

#### Epidermis

The epidermis of the skin was formed of keratinocytes and non-keratinocytes (Fig. 1). In both groups, the keratinocytes are made up of stratum basale, single to several layers of stratum spinosum, stratum granulosum, and stratum corneum. Both the thickness of the epidermis and the number of keratinocytes showed a significant increase in the treated group compared with the control one (Table 2). In addition, the non-keratinocytes as Merkel and Langerhans cells showed a significant increase in their number in the treated group compared with the control one (Table 2). Merkel cells were located between the cells of stratum basale (Fig. 1G). Melanocytes showed non-significant increase in the treated group (Table 2). They contained dark brown pigments were distributed near the basement membrane at the basal cell layer (Fig. 1B-H). However, Langerhans cells were demonstrated on a more superficial position between the cells of the stratum spinosum (Fig. 1I).

**Figure 2:**
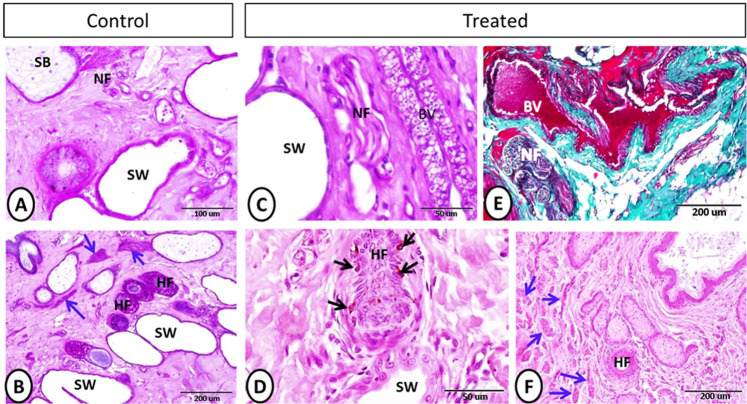
Neuronal elements after the melatonin treatment. (**A–C**) (PAS-Hx staining) and (**E**) (Crossmon’s trichrome staining**)** showing nerve fibers (black arrow), sebaceous gland (SB), sweat gland (SW), and blood vessel (BV). (**B**) (PAS-Hx) and (**D–F**) (Hx-E) staining showing the hair follicles (HF), arrector pili muscle (blue arrows), melanocytes (black arrows) and sweat gland (SW).

#### Dermis

In the dermis of the control and treated groups, the blood vessels of different sizes were observed while the largest one could be seen in a deeper position within the dermis (Fig. 1C,D). Notably, the cross-sectional area of blood capillaries showed a significant increase in the melatonin group compared with the control one (Table 2). On the other hand, both groups showed numerous blood vessels of special structures. In the control group, glomus cells were observed on the tunica media of some arteries. In addition, a vein with a special structure characterized by an irregular lumen and a muscular media of different thickness along the wall was demonstrated (Fig. 1E,F). In the treated group, arteries of special type were characterized by a wide irregular lumen and some of these arteries showed smooth muscle media of different thickness along the wall (Fig. 1J,K). Furthermore, arterioles with special tunica media made up of glomus cells were identified in the melatonin group (Fig. 1L).

Few nerve fibers were demonstrated in the dermis of the control group (Fig. 2A). However, in the treated group, melatonin played in nerve fibers stimulation via increasing the cross-sectional area of nerve fibers significantly, which were observed near the blood vessels, glands, and on a deep dermal position (Fig. 2C–E, Table 2).

**Figure 3:**
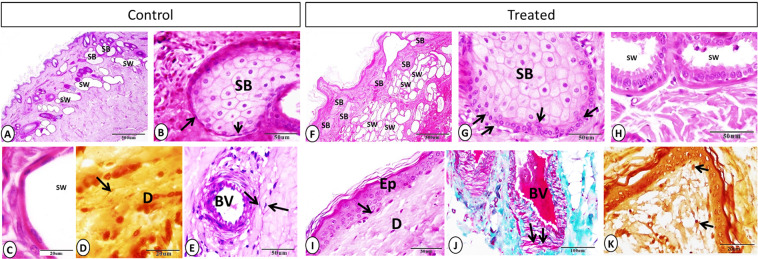
Sebaceous gland, sweat gland, telocytes, and dendritic cells on control and treated groups. (**A–F**) Sebaceous gland (SB) showing hypertrophy and hyperplasia in treated group, and the sweat gland (SW) were more numerous in the treated group (**A**: PAS-Hx. **F**: Hx-E). (**B–G**) Hx-E staining showing sebaceous gland (SB) and basal cells (black arrows), which appeared larger and arranged on several layers on the treated group. (**C–H**) Hx-E staining showing sweet gland (SW) with well-developed secretory activity in melatonin group in compared with control one. (**D-E-I-J**) Telocytes (black arrows), blood vessel (BV), epidermis (Ep) and dermis (D). (**D**) Grimelius silver nitrate method. (**E**) PAS-Hx. (**I**) Hx-E. (**J**) Crossmon’s trichrome staining). (**K**) Grimelius silver nitrate method showing dendritic cell on dermis of treated group (black arrows).

Well-developed arrector pili muscles were observed in the treated group compared with the control one (Fig. 2B–F). In addition, in the treated group, obvious hypertrophy and hyperplasia were detected in the sebaceous glands in comparison with the control group. Furthermore, the basal cells of the sebaceous glands were cuboidal and arranged in 1–3 layers on the treated group. On the other hand, the basal cells of the sebaceous glands of the control group appeared flattened and arranged in 1–2 layers (Fig. 3A–B–F–G). Apocrine sweat glands of the treated group showed a significant increase in their number and diameter with well-developed secretory activity compared with the control one (Fig. 3A-B-C-H, Table 2).

**Figure 4:**
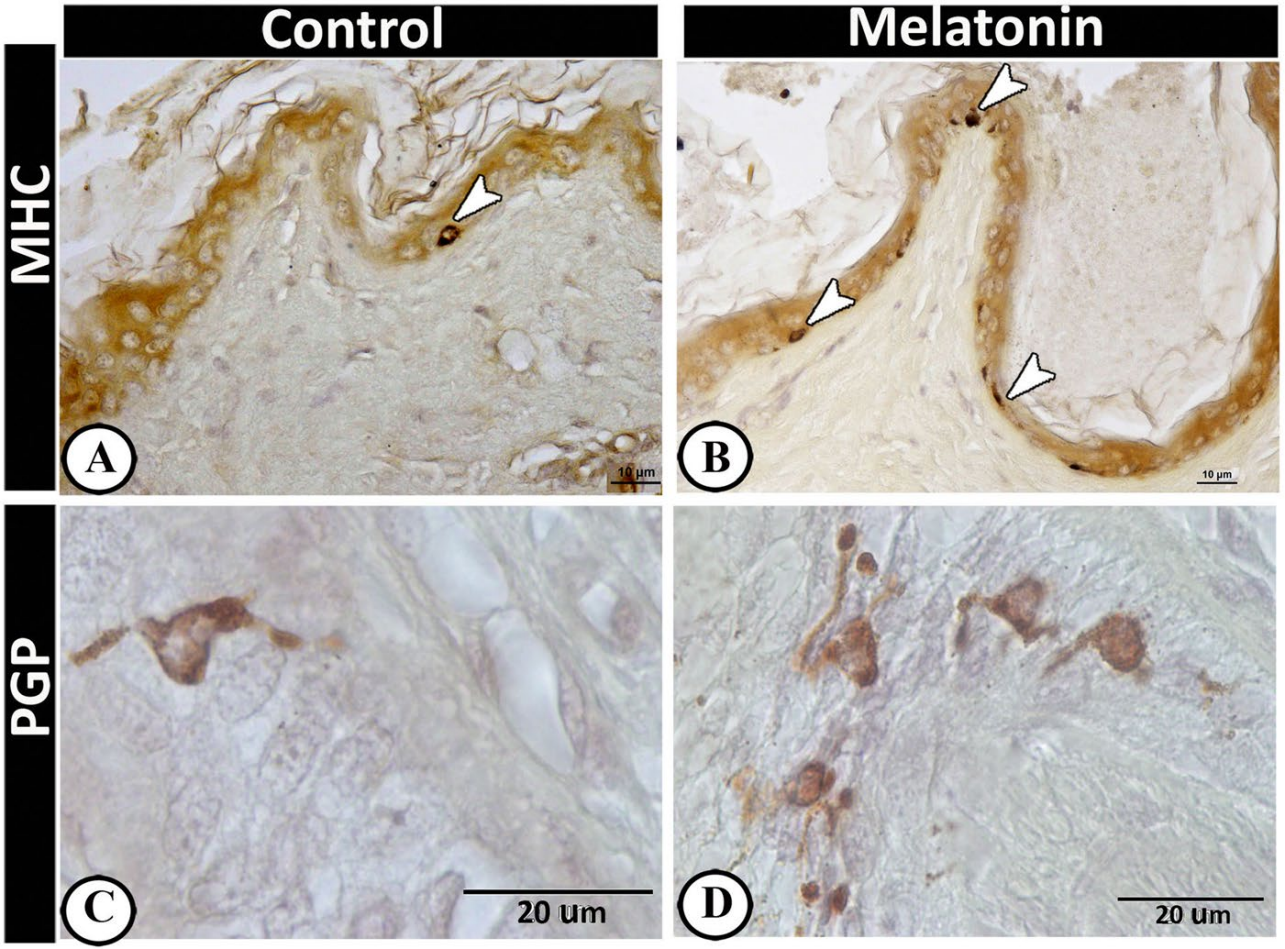
Langerhans cells in the control and melatonin treated groups using MHC II (**A & B**) and PGP (**C & D**) immunoreaction. Langerhans cells (arrowheads) were observed within the epidermal layer and showed high frequencies in the treated group.

Telocytes (TCs) were observed in both the control and melatonin-treated groups within the dermis in close apposition to the epidermis and near the blood vessels (Fig. 3D-E-I-J). TCs were characterized by a small spindle or triangular cell body and long telopodes. On the other hand, the dendritic cells demonstrated on the melatonin treated group on the dermis and near the epidermis. Dendritic cells were characterized by a small cell body and shot cytoplasmic processes (Fig. 3K).

### II-Immunohistochemical analysis

**Figure 5:**
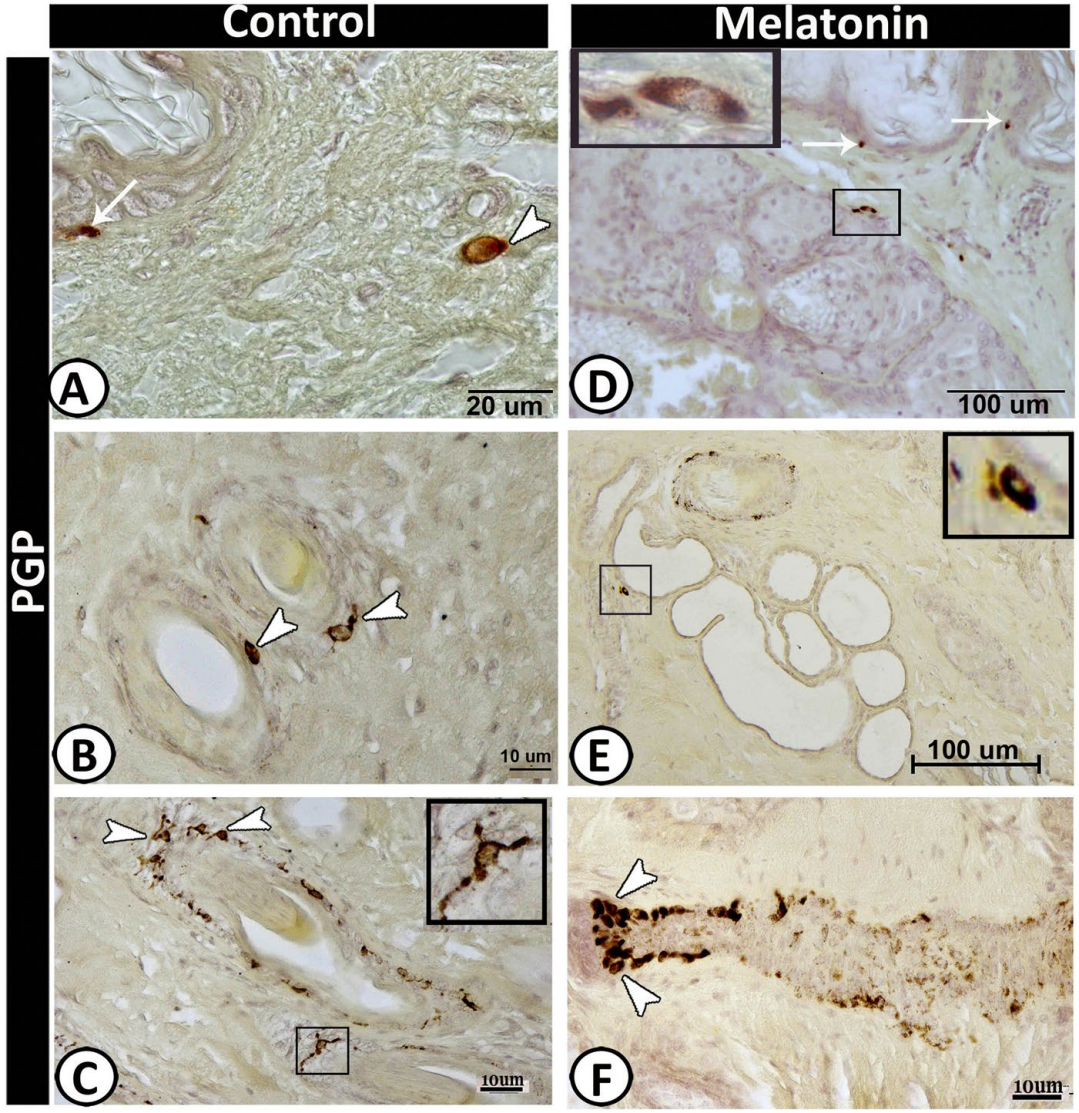
PGP 9.5 immunoreactive cells in scrotal skin in control (**A–C**) and melatonin treated group (**D–F**). Langerhans cells (arrows) were observed in the basal layer of epidermis which were much frequent in melatonin group (**D**) than control (**A**). Dendritic cells (arrow) in the dermis in control group and around the hair follicle (**B & F**), the sebaceous gland (**D**) as well as sweat glands (selected area in **E**). Notice, TCs around the outer root sheath of the hair follicle (selected area in C).

**Figure 6:**
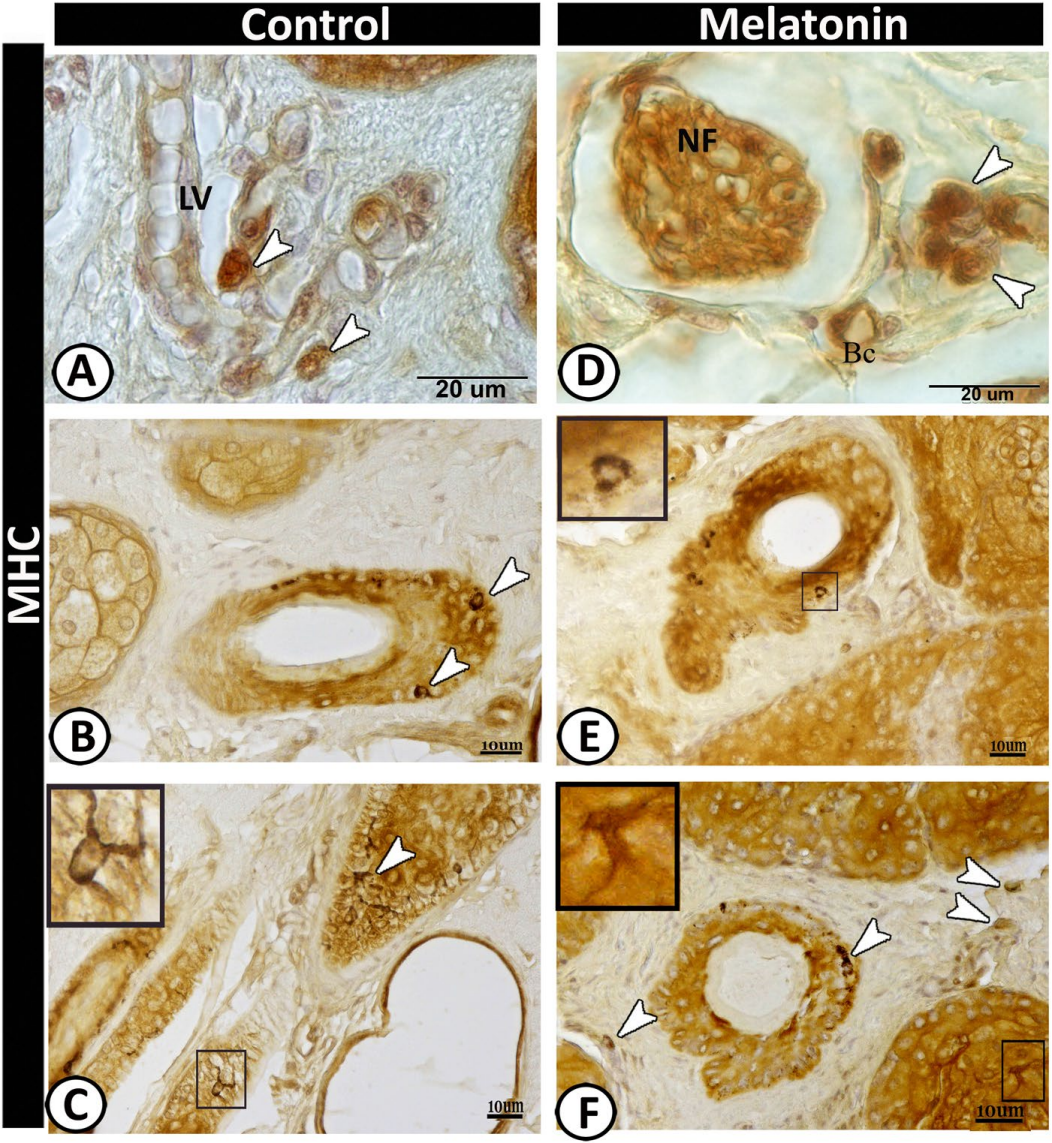
Immunoreactivity of scrotal skin for MHC II protein in control (**A-C**) and melatonin treated groups (**D-F**) showing positive immunoreaction of some of antigen- presenting cells (arrowheads) around the lymph vessel (LV) in control group (**A**) and nerve fiber (NF) in melatonin treated group. Notice, blood capillary (BC). Dendritic cells (arrowheads) around the hair follicles (**B & E**) and scattered in the connective tissue of the dermis in melatonin treated group (**F**). TCs were detected within the outer sheath cells of the hair follicles (selected area in C&F).

**Figure 7:**
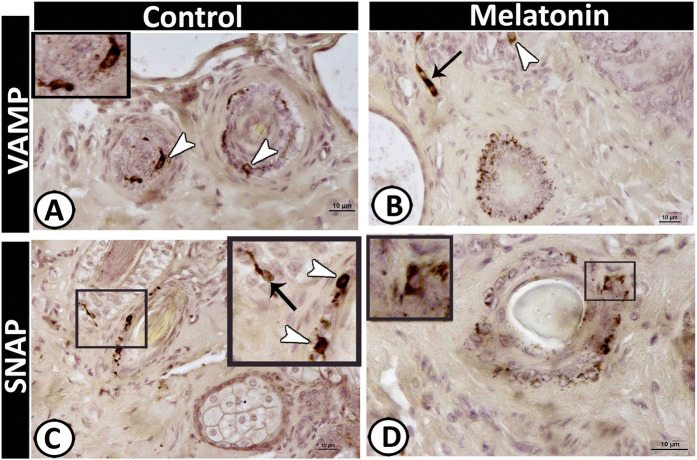
Immunoreactivity of scrotal skin for VAMP protein (**A & B**) and SNAP (**C & D**). Dendritic cells (arrowheads) and TCs (black arrows) showing positive immunoreactivity for both antigens.

**Figure 8:**
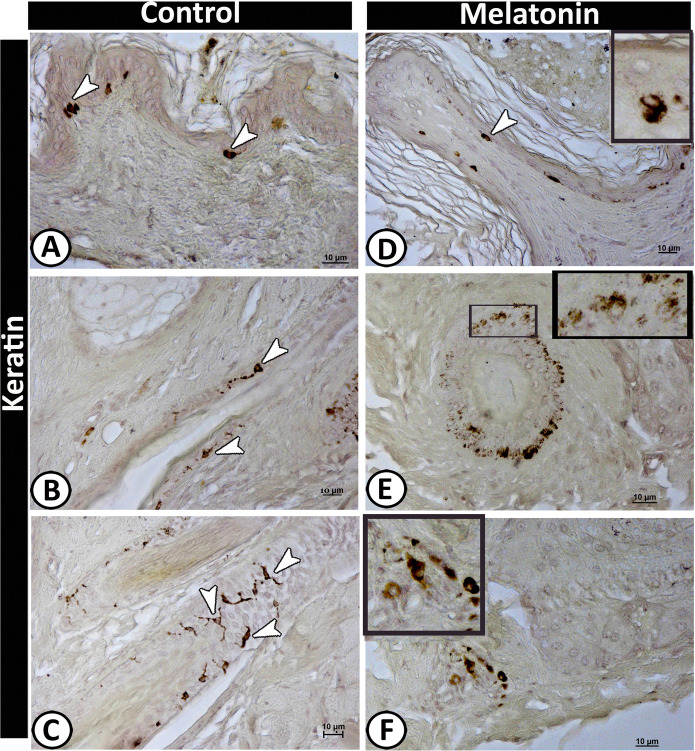
Immunoreactivity of scrotal skin for keratin protein-5 in control (**A-C**) and melatonin treated groups (**D-F**) showing; positive-stained basal active keratinocytes (arrowheads) (**A & D**). TCs (arrows) around the hair follicle showing a positive reaction in the control group (**B & C**). E&F: Aggregation of dendritic cells (selected area) around the hair follicle and some of them scattered in the dermis in melatonin treated group.

The cellular components of the scrotal skin displayed different immunoreactivity in both the control and treated groups. The Langerhans cells showed a significant increase in the melatonin groups (Table 2) that were identified by MHC II (Fig. 4A,B) and Protein gene product 9.5 (PGP 9.5) (Fig. 4C,D) within the epidermal layer. Furthermore, a high numbers of dendritic cells were identified in the melatonin group in compared with the control one (Table 2), which were distributed within the dermis, around hair follicles, sebaceous glands and sweat glands and were strongly expressed PGP 9.5 (Fig. 5), MHC II (Fig. 6), VAMP (Fig. 7A,B), SNAP (Fig. 7C,D), and keratin-5 (Fig. 8) immunoreactivity. The basal active keratinocytes expressed keratin-5 in the melatonin group more than those in the control one (Fig. 8A,D).

**Figure 9:**
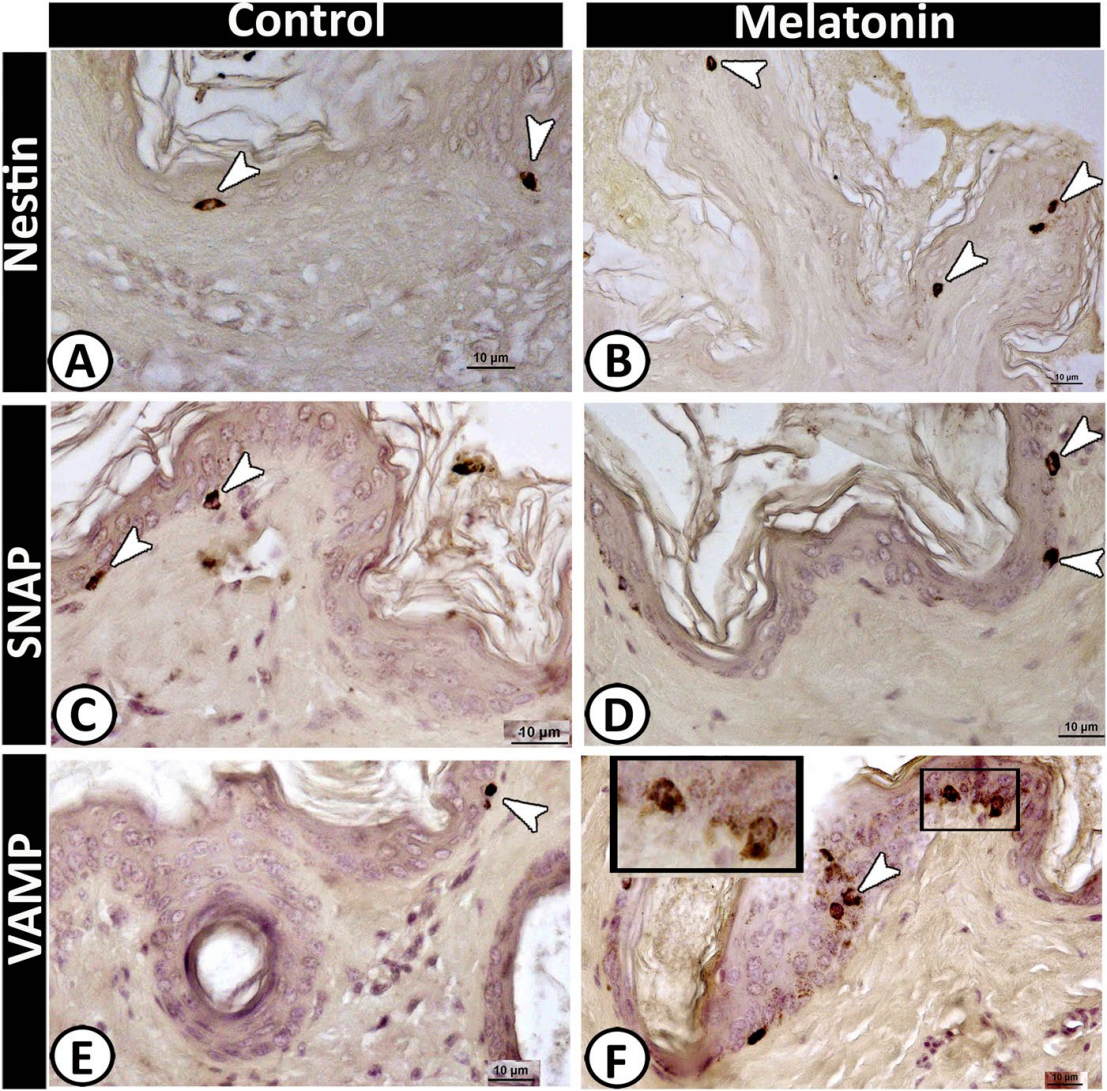
Merkel cells with typical morphology within the basal layer of the epidermis expressed immunoreactivity against Nestin (**A & B**), SNAP (**C & D**) and VAMP (**E & F**).

**Figure 10:**
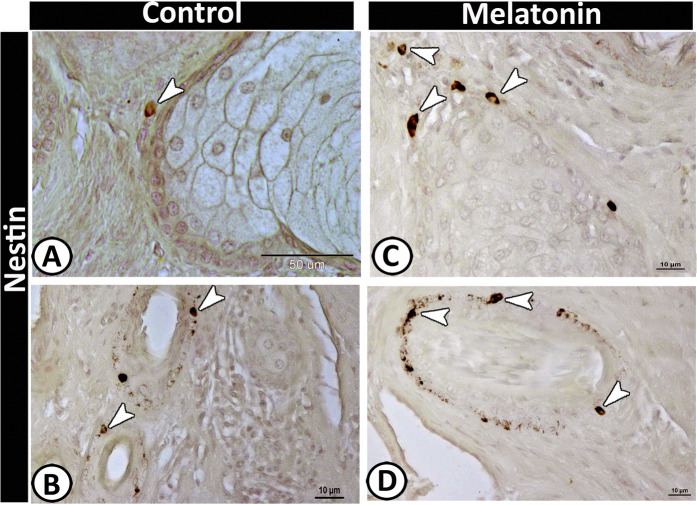
Nestin positive stem cells around the sebaceous gland (**A & C**) and hair follicle (B&D).

Notably, Merkel cells showed a significant increase in the number in the melatonin group (Table 2) that could be stained against the following antibodies; Nestin (Fig. 9A,B), SNAP (Fig. 9C,D), and VAMP (Fig. 9E,F). These cells were identified within the basal layer of the epidermis and around the hair follicles. Positive-Nestin immunoreactive stem cells could be identified clearly in the melatonin group around the sebaceous glands and hair follicles (Fig. 10).

**Figure 11:**
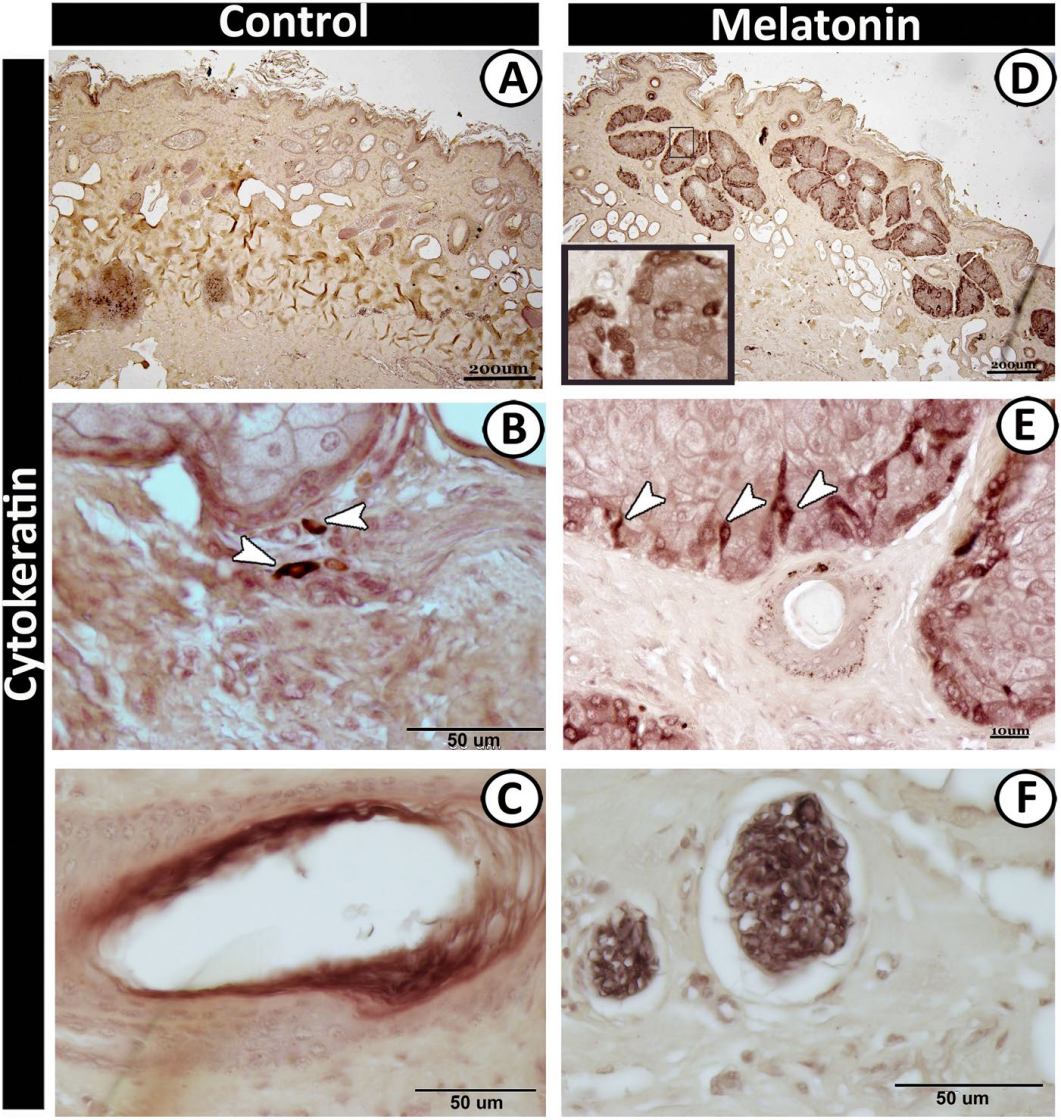
Immunoreactivity of scrotal skin for cytokeratin protein-19 in control (**A–C**) and melatonin treated groups (**D–F**) showing; intense positive immunoreactivity of sebaceous glands in melatonin treated group (**D**) while those of control group (**A**) displayed negative immunoreaction. Strong immunoreactivity of the outer cells (arrowheads) of sebaceous glands (**E**), vascular elements (**C**) and nerve fibers (**F**) in melatonin treated group. Notice, the dendritic cells (arrowheads) exhibiting positive reaction for cytokeratin around the sebaceous glands in control group (**B**).

**Figure 12:**
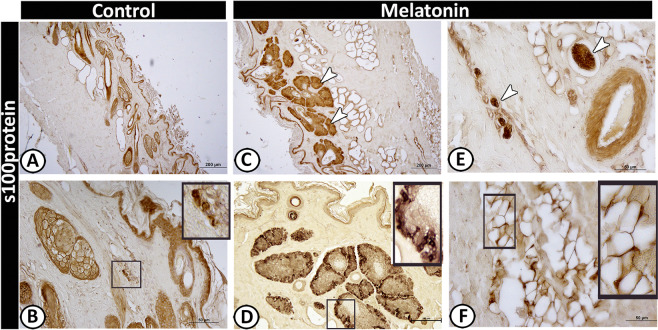
Immunoreactivity of scrotal skin for S100 protein in control (**A & B**) and melatonin treated groups (**C–F**) showing weak positive reactivity of epidermal cell layers in both groups (**A–D**). However, the sebaceous glands (**C,D**), nerve fibers (**E**) and TCs (**F**) showed strong reactivity in melatonin treated group than the control.

**Figure 13:**
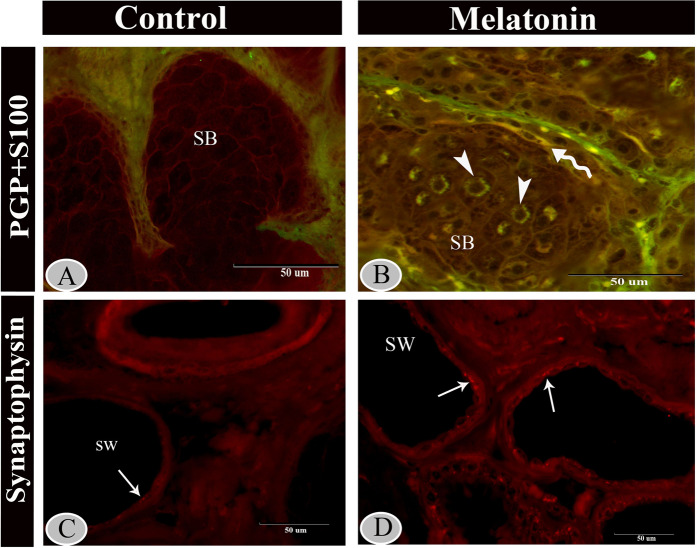
Immunofluorescence staining of scrotal skin in control (**A & C**) and melatonin group (**B & D**). Merged immunofluorescence for S100 (green) and PGP (red) in control group (**A**) melatonin group (**B**), inner cells of sebaceous glands (arrowheads) in melatonin treated group showing positive staining for S100 (green). Notice, colocalization of PGP and S100 (yellow) was exhibited in TC (wavy arrow) which surrounding the sebaceous gland in melatonin treated group. Immunofluorescence for synaptophysin in the sweat gland showing strong positive reactivity of the secretory granules in the melatonin group (**D**) incomparable to the control (**C**).

Moreover, the basal cells of the sebaceous glands were stained with cytokeratin-19 (Fig. 11D,E) and S 100 proteins (Fig. 12C,D) in the melatonin treated group than the control group (Figs. 11A–C and 12A,B) respectively. Furthermore, double immunolabeling of the sebaceous glands showed that some of the inner cells of sebaceous glands exhibited significant immunoreactivity for S100 protein in melatonin treated group (Fig. 13A,B). The nerve fibers were intensively immunoreacted for cytokeratin -19 (Fig. 11F) and S 100 proteins (Fig. 12B,E). On the other hand, the secretory granules in sweat glands were exhibited a strong positive reactivity for synaptophysin in the melatonin group incomparable to the control group (Fig. 13C,D).

**Figure 14:**
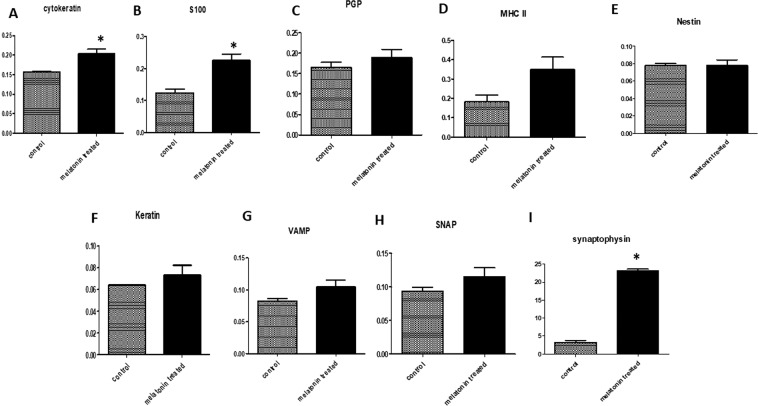
Immunohistochemical (**A-H**) and immunofluorescence (**I**) staining quantification using image j software. A: showing the mean immunohistochemistry staining intensity of the Cytokeratin (**A**), S100 (**B**), PGP (**C**), MHC II (**D**), Nestin (**E**), Keratin (**F**), VAMP (**G**) and SNAP (**H**) is significantly higher in melatonin treated groups than control groups. I: showing the mean fluorescent density of synaptophysin (red puncta) are significantly higher in melatonin treated groups than control groups. The melatonin treated groups is compared to control groups using unpaired t-test (n = 3). *P ≤ 0.05.

In the current work, a high number of TCs could be identified in the treated group around the hair follicle using PGP 9.5 (Fig. 5B) or within the hair follicles by keratin-5 immunostaining (Fig. 8C). Additionally, they were visualized scattered in the dermis around the nerve fibers and blood vessels with S 100 protein (Fig. 12F). For more identification, we used double immunolabeling for S100 and PGP 9.5, TCs showed colocalization of both antigens around the sebaceous gland in the melatonin treated group (Fig. 13A,B).

Finally, the quantitative results of the immunohistochemical and immunofluorescence studies were represented in Fig. 14. Cytokeratin and S100 proteins showed a significantly increase in melatonin treated groups comparable to control groups. Moreover, there was non-significant increase of PGP, MHC II, Nestin, Keratin, VAMP and SNAP in melatonin treated groups than control groups. Additionally, Synaptophysin immunofluorescence within the sweat gland was significantly increased in melatonin treated groups in contrast to control ones.

## Discussion

To our knowledge, the present work is considered the first description of the effect of melatonin administration on the mammal’s scrotal skin. Using some immunohistochemical markers for the first time in this study to identify specific cellular components in the skin. The current study showed that the administration of melatonin induced a stimulatory effect on keratinocytes, non-keratinocytes, sebaceous and sweat glands, hair follicles, and arrector pili muscle. Moreover, the vascular, neuronal, and cellular constituents of the dermis were stimulated after melatonin treatment. These effects of melatonin on the skin are mediated directly or through receptors^1^. Membrane receptors MT1 were demonstrated in keratinocytes and hair follicles^33^, MT2 were demonstrated in hair follicles, sweat gland, blood vessels, and melanocytes^16,34^, and MT3 which act as a regulator in the skin^35^. In addition, the receptor-independent functions of melatonin in the skin include anti-inflammatory and antioxidant activities.

Regarding the useful effects of UVR as improved vitamin D, long-term exposure may lead to wrinkling, pigmentary changes, and cancer^36^. After exposure to UVR, the expression of MT1 and MT2 melatonin receptors by the skin were increased that indicated the response of melatonin to irradiation^16^. In addition, the administration of melatonin before exposure to UVR enhanced the protective effect^37^. Recently, there is a clear concept that melatonin administration is a key player in the protection and maintenance of skin homeostasis and decrease the effect of aging^1^

Our results indicated that the thickness of the epidermis showed a significant increase after melatonin treatment that was associated with an increase in the number of keratinocytes. These results are supported by Song, *et al*.^38^, who described the role of melatonin in keratinocytes proliferation. In addition, a significant increase in the cross-sectional area of the blood capillaries was observed in the treated group. According to Pugazhenthi, *et al*.^39^ and Soybir, *et al*.^40^, melatonin application induces angiogenesis and increases the number of blood vessels in the skin. Rodella, *et al*.^41^ added that melatonin induces improvement of the vascular cytoarchitecture and has a protective role in the case of vessel dysfunction.

Plenty of the nerve fibers innervate the skin, which carry numerous neuronal mediators stimulated peripherally or centrally^42^. Our results revealed that melatonin administration increases the cross-sectional area of the nerve fibers. Similar findings were detected by Mokhtar, *et al*.^26^ and Hussein, *et al*.^27^ on the adrenal gland. Also, melatonin plays a key role in peripheral nerve regeneration and development of the nervous system via its antioxidant activity^43^

Our results revealed that melatonin administration induces hypertrophy and hyperplasia on sebaceous glands. Moreover, sebaceous gland activity depends on melatonin levels^44^. Sebum, the product of the sebaceous gland, is involved in many functions such as; cleaning the skin, fungicidal agent, control bacterial growth, control water loss and if reabsorbed assist in provitamin D production^45^. Moreover, the secretory activity and the number of sweat glands increased significantly after melatonin administration in our work. It is well known that the sweating plays a key role in heat loss control by sweat evaporation so it protects from overheating^46^. In addition, the thermoregulation is mediated by arrector pili muscle contraction, which induces air trapping^47^.

The distribution and antigen expression of the cellular elements of the scrotal skin were identified in the current study. Merkel cells (Mc) are considered as neuroendocrine cells, which located at the basal layer of the epidermis and in the hair follicles^48^. In the present study, Mc expressed Cytokeratin 19, VAMP, SNAP and Nestin. Previous studies have demonstrated that the intermediate filaments of Merkel cells are of the simple-epithelial cytokeratin (CK) type (CKs nos. 8, 18 and 19)^49–51^. Various proteins like VAMP 1/2 and SNAP were first detected in the nervous system as well as non-nervous tissue^52^. Epidermal Merkel cells exhibited characteristics of sensory receptor cells^53^ and make “synapse-like” contacts^54^.

Langerhans cell is one of the dendritic and antigen-presenting cells, which express both MHC I and MHC II molecules, uptake antigens from the skin and transport them to the lymph node^55^. This comes in agreement with our study, which revealed the expression of MHC II in all dendritic cells including Langerhans cells within the epidermis and other dendritic cells that scattered within the dermis. Furthermore, the current results indicated that melatonin increases the expression of MHC II in dendritic cells. These results matched with findings of Brazao, *et al*.^56^ who found that melatonin enhances the expression of MHC II in antigen-presenting cells. In addition, the present study showed that the dendritic cells can express neuronal proteins, such as Protein gene product 9.5 (PGP 9.5). PGP 9.5 might be related to the maturation of dendritic cells. Moreover, PGP 9.5 is the most specific neuronal protein in the epidermis^57^ that involved in modulation of some membrane receptors, regulation of cell growth, and turnover of some cytoskeletal elements.

Melatonin is the major neuroendocrine regulator, which translates the photoperiod changes to complicate the endocrine responses^58,59^ that in turn may affect the mammalian skin physiology. In the current work, the numbers of Merkel and dendritic cells were increased significantly in the melatonin treated group than in the control one. These findings may be attributed to the role of melatonin in the enhancement of skin immunity, which is regulated by the local neuroendocrine system through the action of locally produced hormones, neurohormones, neurotransmitters, and cytokines^34^.

The current quantitative analysis of keratin-5 showed that melatonin increases the expression of the skin’s keratins. These results are supported by Kim, *et al*.^60^ who reported that exogenously administrated melatonin enhances the skin barrier and wound healing. Furthermore, the present study showed that melatonin induced the expression of nestin, the intermediate filament protein, in stem cells. These results indicated the role of melatonin in skin regeneration.

Telocytes are interstitial cells, which have been demonstrated in many tissues such as the brain, bone marrow, pancreas, lungs, and eyes^61–64^. The present study distinguished TCs by PGP 9.5, S100, cytokeratin-19, keratin-5 and MHC II around the hair follicles and sebaceous glands. Popescu, *et al*.^65^ supposed that TCs may be considered as stromal progenitor cells that can affect the activity of surrounding cells through paracrine signaling. Most nerve fibers were observed in contact or in the vicinity with TCs that indicated the function of the TCs in neurotransmission. Popescu, *et al*.^63^ added that the TCs are involved in the exchange of genetic information with the immune cells and nerve fibers or in sensing changes in the stromal microenvironment

Furthermore, melatonin can regulate cutaneous adnexal function; melatonin receptor (MT2) is found in the adnexal structures^66^. This is in concordance with the current study, which revealed that the cells of the sebaceous gland were strongly positive for s100 proteins and cytokeratin-19 in the melatonin treated group than in the control. In addition, the present study showed intense synaptophysin immunofluorescent granules in sweat glands in the melatonin treated group. The presence of synaptophysin in the skin has pronounced neuroendocrine effects and neuroimmuno-endocrine properties^67^. That may attribute to a thermoregulatory effect of melatonin specifically in healthy males^67^. Melatonin also regulates hair pigmentation and growth^66^.

## Conclusion

The present study showed that melatonin administration caused a stimulatory action on all cellular, glandular, vascular, and neuronal components of the skin. Specifically, the number of Merkel, Langerhans, and dendritic cells showed a significant increase after melatonin treatment. Melatonin increases the expression of synaptophysin, Cytokeratin, S100, MHC-II, Nestin, keratin, etc. Melatonin may be attributed to many functions in the skin. Therefore, further research should be done to explore how melatonin administration can be therapeutically targeted in future clinical dermatology.

## Data Availability

The datasets used and/or analyzed during the current study are available from the corresponding author on reasonable request.
